# Confinement effects and acid strength in zeolites

**DOI:** 10.1038/s41467-021-22936-0

**Published:** 2021-05-11

**Authors:** Emanuele Grifoni, GiovanniMaria Piccini, Johannes A. Lercher, Vassiliki-Alexandra Glezakou, Roger Rousseau, Michele Parrinello

**Affiliations:** 1 Department of Chemistry and Applied Biosciences, ETH Zurich, c/o USI Campus, Via Giuseppe Buffi 13, Lugano, Ticino Switzerland; 2 Institute of Computational Science, Università della Svizzera italiana (USI), Via Giuseppe Buffi 13, Lugano, Ticino Switzerland; 3 Institute for Integrated Catalysis, Pacific Northwest National Laboratory, Richland, WA USA; 4 Department Chemie and Catalysis Research Center, TU München, Lichtenbergstr. 4, Garching, Germany; 5 Italian Institute of Technology, Via Morego 30, Genova, Italy; 6 Present Address: Scuola Normale Superiore, Piazza dei Cavalieri, Pisa, Italy

**Keywords:** Heterogeneous catalysis, Thermodynamics, Molecular dynamics

## Abstract

Chemical reactivity and sorption in zeolites are coupled to confinement and—to a lesser extent—to the acid strength of Brønsted acid sites (BAS). In presence of water the zeolite Brønsted acid sites eventually convert into hydronium ions. The gradual transition from zeolite Brønsted acid sites to hydronium ions in zeolites of varying pore size is examined by ab initio molecular dynamics combined with enhanced sampling based on Well-Tempered Metadynamics and a recently developed set of collective variables. While at low water content (1–2 water/BAS) the acidic protons prefer to be shared between zeolites and water, higher water contents (*n* > 2) invariably lead to solvation of the protons within a localized water cluster adjacent to the BAS. At low water loadings the standard free energy of the formed complexes is dominated by enthalpy and is associated with the acid strength of the BAS and the space around the site. Conversely, the entropy increases linearly with the concentration of waters in the pores, favors proton solvation and is independent of the pore size/shape.

## Abstract (web summary)

The structure of water around Brønsted acid sites in zeolites is shown to influence their catalytic activity. Here the authors shed light on confinement effects in different pores zeolites/water interfaces acidic strength by means of ab-initio molecular dynamics and enhanced sampling metadynamics techniques.

## Introduction

Brønsted acid catalysis is one of the most important classes of chemical conversion^1–11^ and is critical for the reactions in petroleum refining and petrochemical industry, including for example hydrocarbon cracking^12–16^, alkylation^17^ and oligomerization, dehydration of alcohols^18–22^, aldol condensation^23–25^, ketonization^26^, and esterification^27,28^. A wide variety of solid acids are used for such reactions with zeolites being one of the dominating groups^4,29,30^.

**Fig. 1:**
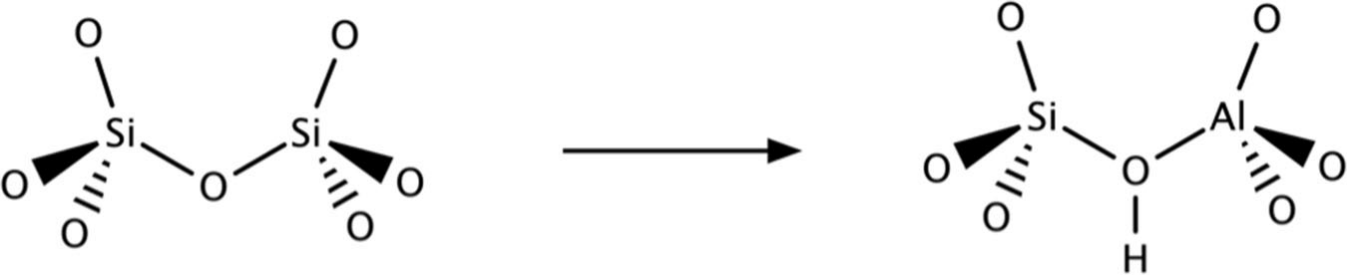
Schematic reaction of brønsted acid site formation. BAS is obtained by replacing a Si^4+^ atom in the zeolite frame with a trivalent metal.

Zeolites are tectosilicates with an impressive number of potential ways to link the corner shared SiO_4_ tetrahedra forming a wide variety of pores and cavities^31–33^. Some of these framework tetrahedra are synthetically exchanged for other elements than Si^4+^, typically Al^3+^, but also Ga^3+^, B^3+^, Ge^4+^, and Ti^4+^. A charge imbalance is created, if the metal cation has a 3+ oxidation state. The resulting negative charge of −1 in the tetrahedron with oxygens is balanced by metal cations or H^+^. The proton is stabilized as an OH group on one of the four oxygen atoms linking the tetrahedron to a neighboring one. It may fluctuate its position among the four oxygen atoms. This results in a “bridging” OH group with the potential to act as a Brønsted acid site (BAS) (Fig. 1).

In its water free state, the ability of a BAS to protonate a base has been studied experimentally and theoretically to an impressive extent^5,34,35^. While early studies had suggested that zeolites would act as super acids^36^ and show widely varying acid strength, it was found that the pK_a_ was lower than that of super acids and depended moderately on the crystal structure. Modern experiment and theory have shown conclusively that zeolites possess a high acid strength, but are relatively insensitive to structural effects and are mildly sensitive to the chemical composition^7,37^. It has been shown, however, that the constraints of the pores stabilize transition states of reacting molecules in a way that these materials show higher catalytic rates compared to counterparts of equal chemical composition on flat or mesoporous surfaces^6,38,39^.

Aluminum containing zeolites have been shown to be hydrophilic. Purely siliceous materials are highly hydrophobic, i.e., they do not stabilize the continuous sorption of water molecules in a density equivalent or approaching liquid H_2_O, even if engulfed in water^40–42^. The fact that it takes substantial external pressures to force water into zeolite pores of siliceous materials demonstrates that the loss of entropy from bulk to confined water prevents the full utilization of the whole pore volume^43^.

Recently, it was shown that the hydrated hydronium ions sorbed water are limited in size by the difference between the standard free energy of the water cluster in the pores compared to one in the aqueous phase^44,45^. For MFI zeolites with pores ~0.6 nm, this balancing act leads to hydrated hydronium clusters containing approximately eight water molecules.

The catalytic activity of such hydronium ions benefits from the constrained environment, with rates approximately 1–2 orders of magnitude higher than those in an open aqueous environment^46–48^. Organic molecules appear to adsorb in such partly filled pores only in the voids left unoccupied by hydrated hydronium ion clusters. This leads to marked discontinuities in the arrangements of molecules that have been shown to be equivalent to the impact of liquids of high ionic strength^44^. There have been several prior studies of water in zeolites at the classical molecular dynamics^49–52^ (CMD) and AIMD^53–57^ levels of theory. The latter studies, which account for both reactivity and diffusion, reveal a complex behavior of the protonation state as a function of the number of adsorbed water molecules and the nature of the BAS ranging from sharing the proton equally between water and the BAS, to complete solvation of the proton within water clusters. Recently, AIMD combined with IR and high-resolution solid-state NMR on H-ZSM5 zeolites, showed that the interaction between the proton and the water cluster surrounding the catalytic site weakens with increasing water loading and plateaus upon adsorption of 7–8 water molecules per BAS^44,45^. A preliminary computational study performed with AIMD simulations and static DFT calculations supported these conclusions^58^, and showed that the bond distance between the acidic proton and the releasing framework oxygen is proportional to the water concentration.

Understanding and controlling the impact of water ordering in zeolite pores leads to a better understanding of the hydrophobicity and allows us to predict and model the elementary steps of acid catalyzed reactions in confined spaces. This understanding requires, however, addressing questions related to: (i) the nature and thermodynamics of solvation of the BAS in the presence of increasing chemical potentials of water and (ii) the size of hydrated hydronium ion clusters.

In this work, we examine these questions by a combination of ab initio molecular dynamics (AIMD) and enhanced sampling based on the Metadynamics formalisms for studying the rare events dynamics and evaluating the associated free energy landscapes. Here, we ask if this formation of water clusters for proton solvation is an attribute of the MFI structure or if it is a generic property of all zeolites and their cavities. We focus on the role of confinement on the specific population of water clusters and assess the enthalpic and entropic contributions to the zeolite acid–base equilibrium. Due to the presence of energy barriers separating different protonation states, a standard simulation cannot fully address these questions. In order to overcome these limitations, we performed full ab initio Metadynamics simulations^59–61^ to assess the relative free energies of potential protonation sites. Moreover, in ab initio simulations a balance must be found between accuracy and computational efficiency. The latter will determine the thoroughness of the sampling. Here, we have put more weight on this second aspect of the calculation given the highly fluctional character of the hydronium behavior and the fact that we are more interested in statistical distribution rather than individual static structures. A good compromise between accuracy and efficiency for absorbed system on the catalytic interface with oxides is represented by the PBE functional including semi-empirical dispersion by Grimme^62^. To further asses this choice, we also performed preliminary simulations of water clusters comparing the effects of PBE+D2 and B3LYP+D2 on the radial distribution functions for three cluster sizes, see Supplementary Note 5.

In order to probe changes in acid strength and to determine if a general behavior exists, we use on several zeolite BAS and at different levels of water content two recently developed Collective Variables^58^ (CVs) that perform extremely well in studies of proton dissociation events.

**Fig. 2:**
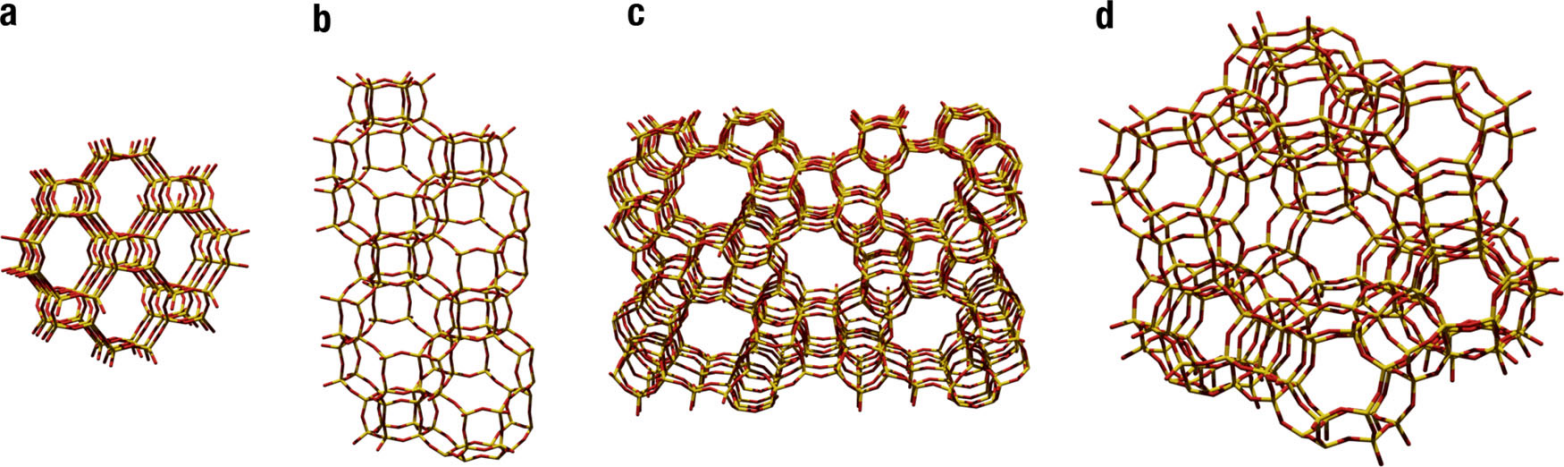
Structures of zeolites employed in this work. **a** GIS, which consists of 8 and 4 ring size cages with diameter 4.97 Å, **b** CHA which consists of 8, 6 and 4 ring size cages with diameter 7.37 Å, **c** MFI which consists of 10, 6, 5 and 4 ring size cages and **d** FAU which consists of 12, 6 and 4 ring size cages with diameter 11.24 Å.

The protonation state of the BAS was studied for four different zeolites^63^, Zeolite Socony Mobil–5 (ZSM-5 or MFI), Chabazite (CHA), Faujasite (FAU) and Gismondine sodium phase 1 (GIS-NaP1), with pore diameters ranging from 0.5–1.5 nm^64^ (Fig. 2). For each zeolite topology we loaded the system around the BAS with an increasing number of water molecules. We, thus, evaluated the energetic and structural properties of these systems comparing topology and water loading. Analysis of the Free Energy Surfaces (FESs) and behavior of the water clusters in contact with the BAS provided us with thermodynamic and mechanistic information with atomistic detail.

## Results and discussion

### Free energy surfaces for proton transfer

In order to understand how the acidic behavior of BAS is altered by the zeolite topologies, reaction free energies have been computed and decomposed to their enthalpic and entropic components. For this purpose, we adopted two recently developed CVs^58^ as are a measure of the protonation state of our systems, s_p,_ and the distance between the charge carrier and the BAS site, s_d_, see Supplementary Note 1 for details.

**Fig. 3:**
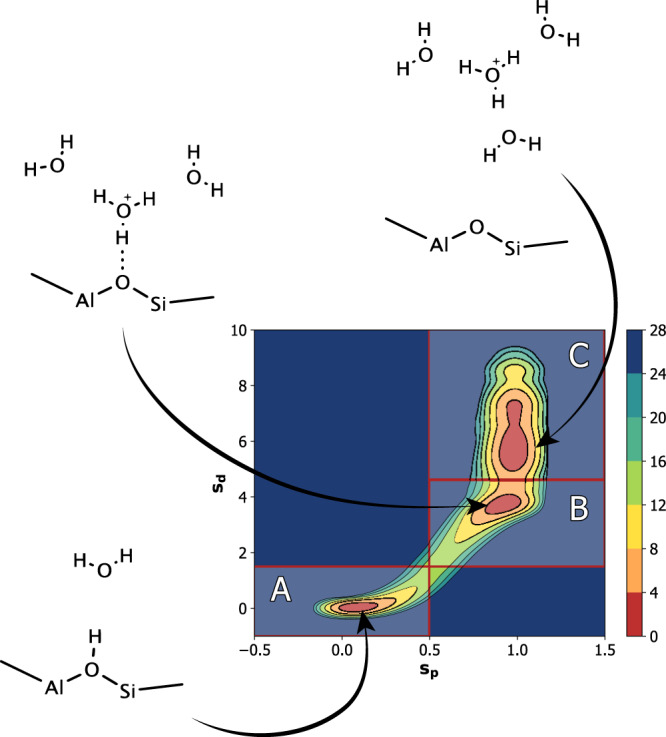
Explicative free energy surface. The FES projected along ${s}_{p}$ and ${s}_{d}$ shows three basins corresponding to the three different configurations that occur during hydronium formation. Colorbar reports free energy differences expressed in kJ mol^−1^.

Figure 3 reports an explicative FES example thus obtained. Its free energy profile clearly presents three well defined thermodynamic states corresponding respectively to the system in its reference undissociated state with the proton covalently bonded the zeolite (A), another one in which the proton jumps to the closest water molecule forming a Zundel-like structure (B) and finally a state in which a hydronium ion is fully solvated and free to diffuse within the cavity (C).

**Fig. 4:**
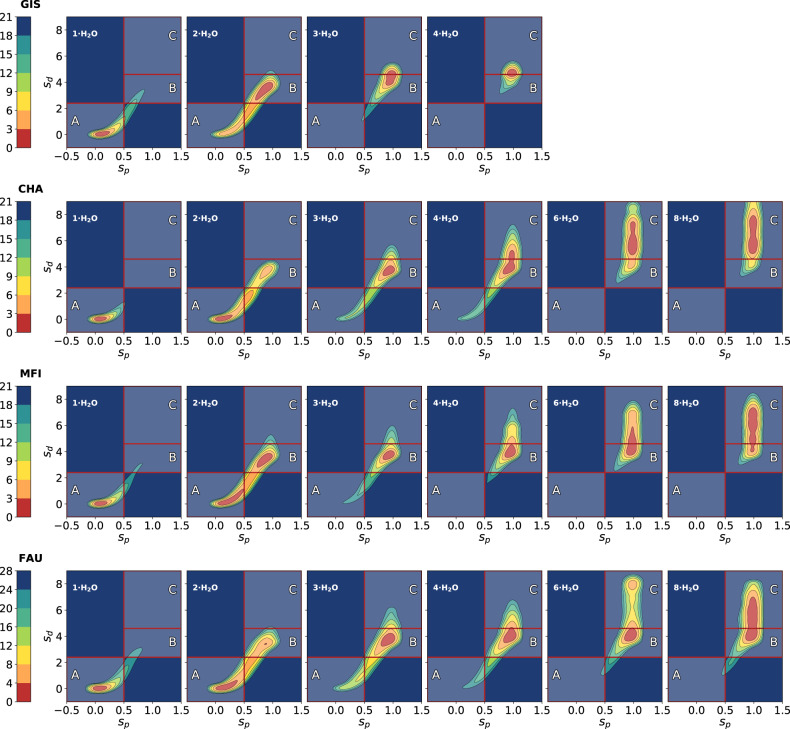
Deprotonation free energy surfaces. These plots report FESs projected along ${s}_{p}$ and ${s}_{d}$ at different hydration levels for the four acidic zeolites. In the case of Gismondine, FES with the cluster made by 6 and 8 water molecules are not present since these systems could not be studied. Differences in free energies are expressed in kJ mol^−1^.

Figure 4 reports the FESs of the four zeolites at different level of water loading. Each FES has been divided in the previously introduced areas and integrated in order to get their relative population. As can be seen, FESs thus obtained have roughly the same shape for all these systems with a deep and narrow minimum for ${s}_{p}$ and ${s}_{d}$ equal to zero and an elongated branch for ${s}_{p}$ equal to one. These states represent the undissociated and dissociated BAS, respectively.

In all these four cases, we observe the same trends as a function of the number of waters. At low water loading (*n* = 1, 2) there is a preference for the proton to remain in the vicinity of the BAS site (s_p_ = 0, s_d_ = 0). However already at *n* = 2 there is finite probability for the proton to move away from that wall by as much as 4 Å (s_p_ = 1, s_d_ = 4). This tendency increases with the number of water molecules, such that by *n* = 8 the proton is completely solvated by water molecule (s_p_ = 1, s_d_ > 4). This is in agreement with previous AIMD studies which show that the proton becomes “solvated” at higher water concentrations, but remains close to the BAS site at low hydration levels^45^.

**Fig. 5:**
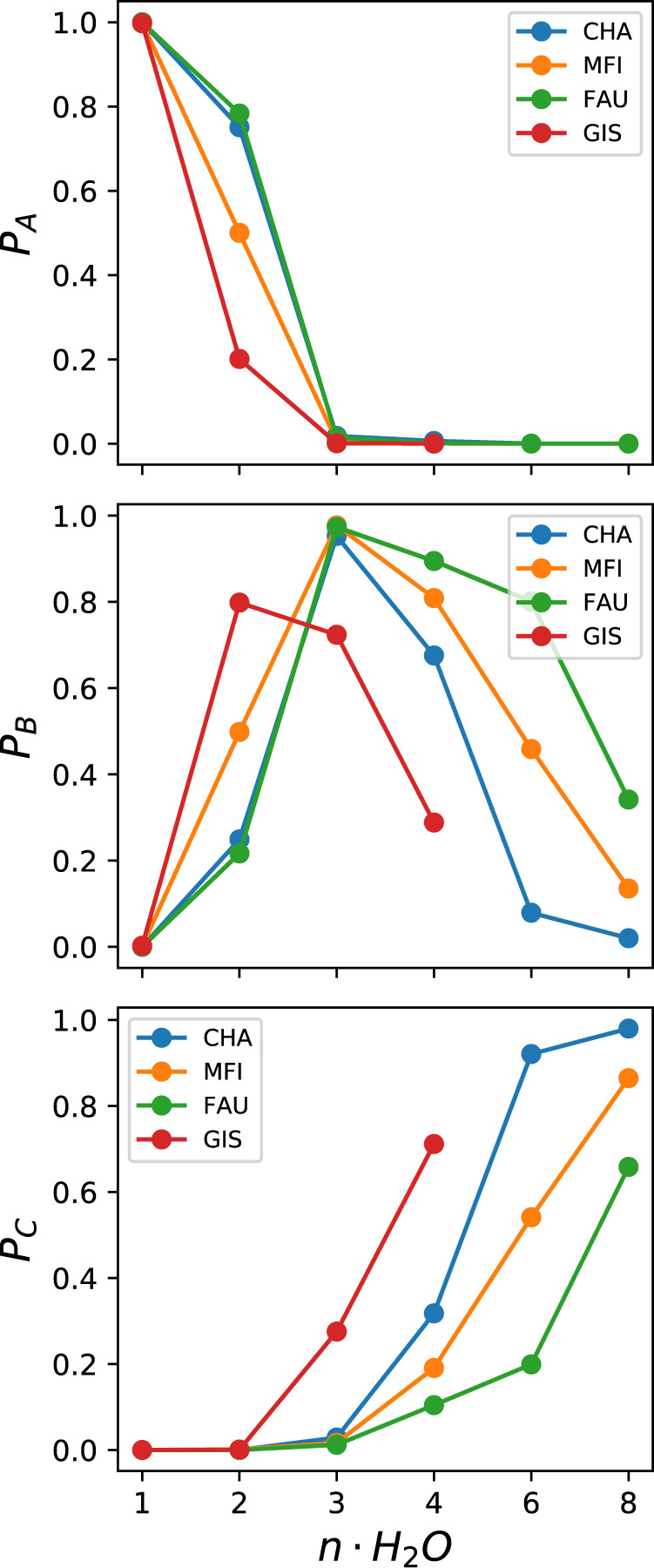
Relative populations as a function of water loading. Relative population of the state A (top panel), B (middle panel) and C (bottom panel) as a function of the water content for every zeolite.

From these populations, we can observe that the same trend of transferring the proton from the wall as a function of hydration level occurs in all four zeolites see Fig. 5. In all cases, minimum A with the proton at the BAS becomes negligible once the water concentration exceeds 2 per BAS. It is minimum B, with the Zundel-like structure, where the most distinction between the zeolite frameworks occurs. Although the population of B shows a maximum at *n* = 3 and drops off with increasing *n*, the rate at which it does so is not the same amongst the different zeolites. Its population decreases more rapidly the smaller the cage. Hence a more careful analysis of the energetics is required to quantitatively understand these trends.

### The role of enthalpy and entropy in determining protonation state

**Fig. 6:**
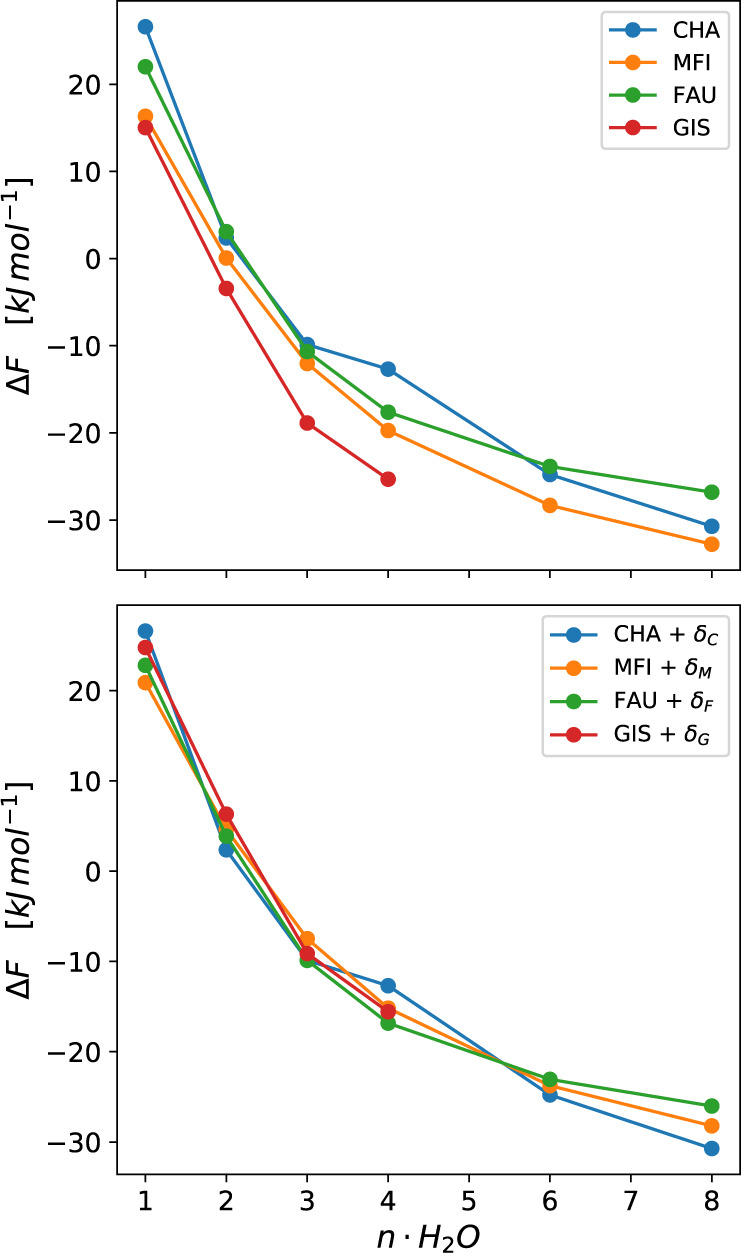
Deprotonation free energies. Reaction free energies between states A and B+C as a function of the water loading (top panel) and same curves rescaled by a factor δ that minimizes the offsets among them (bottom panel).

In order to understand the thermodynamic parameters determining the protonation state of water in zeolites, we use the relative trend in free energy between the undissociated (A) and the hydrolyzed states (B+C) as a function of the water loading. This function shows a universal behavior among different zeolites, see Fig. 6. Most importantly, except for small differences, all systems present the same decay trend with increasing *n*, but are offset at *n* = 1 due to the differing acid strength of the BAS as imposed by the zeolite framework Indeed, shifting vertically all these curves by a constant value. ${\rm{\delta }}$ that compensate these small differences shows that all these curves can be collapsed in one. $\delta$ values used in Fig. 6 are $0,4.56,0.79,$ and $9.75$ for Chabazite, MFI, Faujasite, and Gismondine respectively. This implies that although the free energetics at low hydration level may depend on local structural features (small differences in enthalpy), the behavior at increasing n is being determined by a structurally insensitive free energy term.

**Fig. 7:**
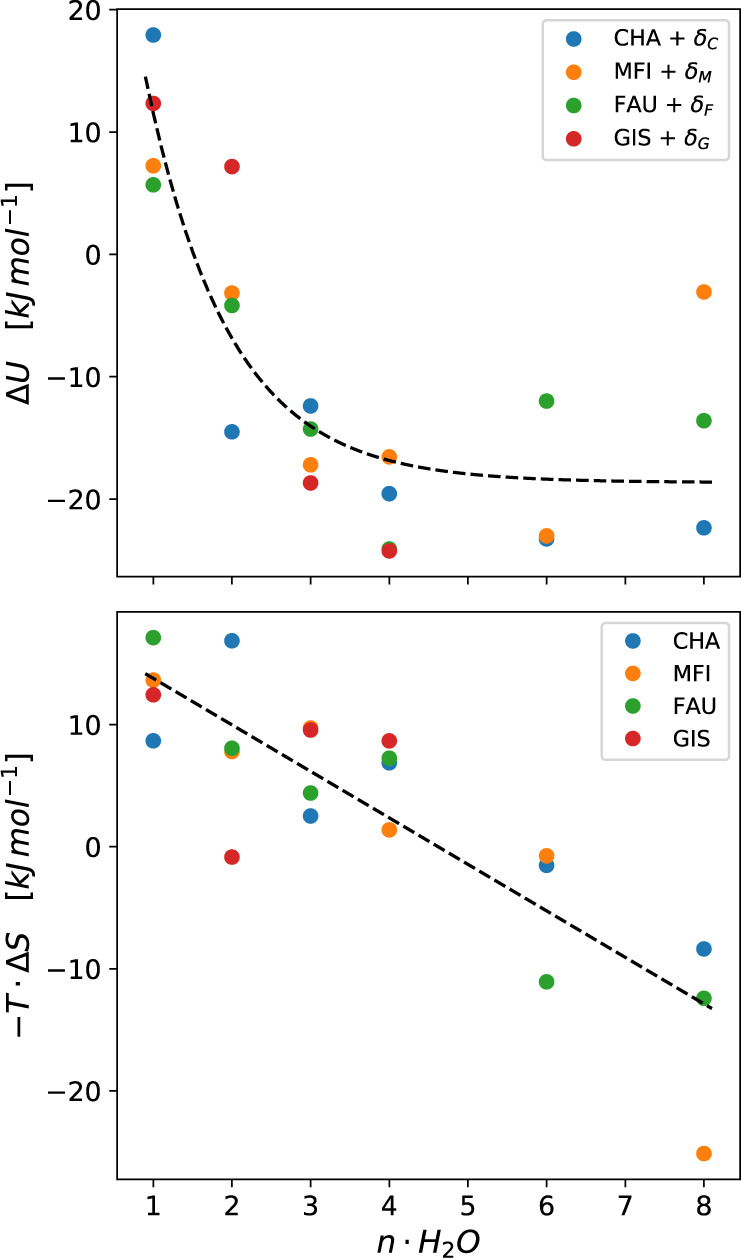
Enthalpic and entropic component decay. Exponential and linear decay of the enthalpic (top panel) and entropic (bottom panel) contributions to the generation of the hydrated hydronium ion.

To further probe this observation, we separate the free energy into contributions by the internal energy ($\Delta U$) and entropy ($\Delta S$)^65^ to determine the driving force for proton solvation. The trends in $\Delta U$ as a function of the number of waters are given in Fig. 7, and shows that in all zeolites $\Delta U$ varies significantly between *n* = 1–3, but tapers off to about 20 kJ mol^−1^ for *n* = 3. This initial fast drop as well as the identical behavior of the four zeolites is attributed to the fact that the hydronium needs a certain number of water molecules to be stabilized, while the framework of the zeolites plays only a minor role. Once the cluster is sufficiently large, further addition of water molecules does not impact the solvation energy of the proton. The result clearly shows that for more than 3 water molecules further association does not lead to a better enthalpic stabilization.

This is further substantiated when one considers the entropy computed (Fig. 7), showing a nearly linear contribution to the free energy with increasing number of water molecules. All zeolites show a similar decrease with a slope of approximately −4.0 kJ mol^−1^/H_2_O regardless of the cavity size, i.e., the entropy increases solely as a function of the number of waters and is not influenced by the cavity size. Thus, we conclude that the main factor for this increase is related with the larger environment in which the proton can be mobile, i.e., the entropy of the proton depends on the size of the hydrogen bond network it is part of and that scales with the volume of the cluster. As the zeolite framework perturbs this property of the clusters very little this entropic stabilization of the proton is, to first order independent of the zeolite lattice.

We note that the above analysis examines the stabilization of proton as a function of the water cluster size which is a leading driver for water adsorption in zeolites. Our analysis indicates that adsorbed water will cluster adjacent to the BAS regardless of the zeolite structure. However, the energy stabilization of the proton (DU) evens out at *n* = 4 and above, therefore the changes in the free energy are the result of the TDS term coming from the delocalization of the proton as the cluster increases. Thus, the thermodynamic drivers for increasing the cluster size becomes attenuated as the heat of adsorption of additional waters within the zeolite pore can no longer compensate for the heat of condensation for water outside of the zeolite. This explains why the cluster do not increase beyond a certain size consistent with our previous experimental observations^44,45^.

### Structure of water clusters within the zeolite framework

**Fig. 8:**
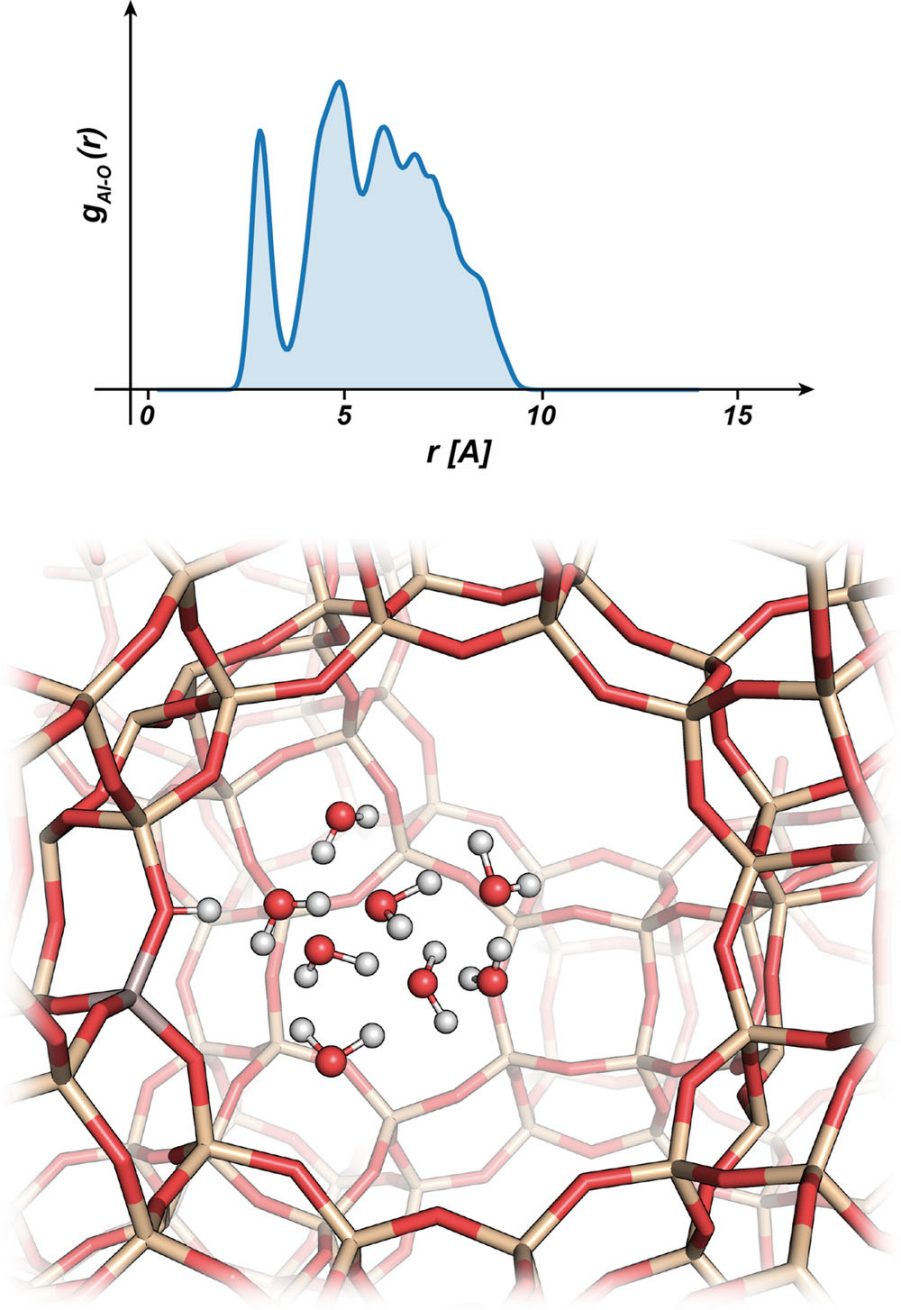
Water cluster arrangement in zeolite pores. Al-O_w_ radial distribution function (top panel) overlaid on a faujasite cavity and its water molecules (bottom panel).

**Fig. 9:**
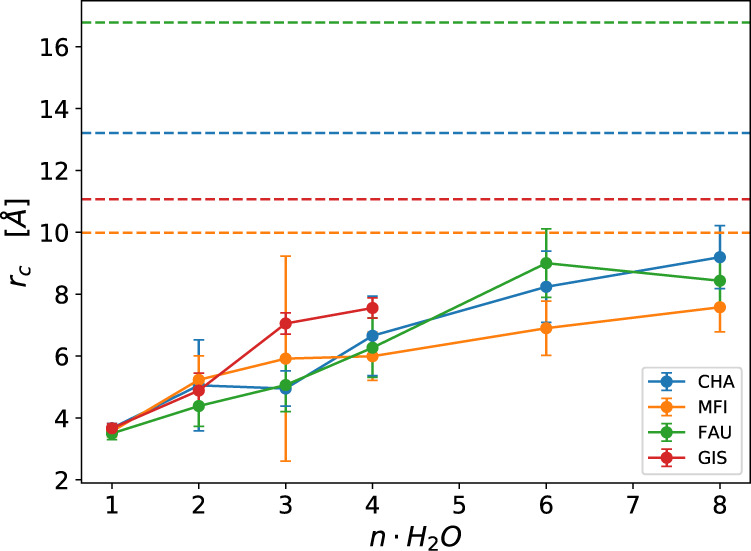
Radii of spheres around the aluminum atoms enclosing the 90% of the water density. Dashed lines report the maximum Al-Si distances in the respective cavities which indicates the approximate diameters of the main channel or pore. Error bars report standard deviations of each distribution.

In order to further investigate how water molecules are arranged in pores, we calculated the water density around a BAS integrating the radial distribution functions correlating the aluminum-oxygen distances, see Fig. 8. For a full report of these data please refer to the Supplementary Note 4. To estimate the portion of volume explored by the clusters for each different zeolite topology upon water loading, we integrated the radial distribution functions and obtained the radii of the spheres containing the 90% of the water clusters around the BAS, see Fig. 9.

Regardless of the zeolite topology, we observed that for the specific water loading considered in this work the solvent molecules spend almost all of their time in a region close to the BAS and the volume increases only upon loading. Therefore, a large fraction of the cavity remains unoccupied suggesting that the confinement effects provided by the zeolite walls exert a negligible effect on the structural behavior of the cluster.

This is caused by the first water molecule remaining close to the zeolite framework (avoiding excessive charge separation) and to the hydrogen bond network that keeps the water clusters compact and prevents waters to diffuse inside the entire cavity. We must note that for the smallest topology considered here (GIS) the thigh channels of the framework limit the water loading around the BAS to a maximum of 4 water molecules. Above this number, the clusters tend to split and diffuse in the neighboring cavities and, therefore, we did not consider higher loadings.

From a statistical mechanics’ point of view, entropy, and more precisely the translational contribution, is proportional to the accessible volume of the system. We also know that the positive charge can move only inside the water network hopping from water to water through the hydrogen bond network. As a consequence, translational entropy grows proportionally with the volume of the water cluster and not necessarily with the zeolite cavity size. This implies that the entropic contribution to proton solvation is independent on the zeolite framework type and does not stem from the mobility of the overall cluster in the available pore. A further analysis showed that the fluctuations of the structure as measured by the surface to volume ratio are not affected by the presence of the walls. In fact, similar behavior is observed in both confined and gas phase clusters (see Supplementary Note 4).

In conclusion, the universal behavior of BAS solvation in a select group of zeolite frameworks was examined by means of ab initio Metadynamics methods. Independently of cavity shape or size, water clusters tend to fully solvate the excess proton starting from loadings higher than 3. The volume of the water clusters and entropy of solvation are independent on the zeolite framework type. As a result, the free energy of proton solvation follows a universal behavior. Enthalpy contributes only very little to the stabilization of the proton from loadings higher than 3, while the entropic contribution grows linearly with the number of water molecules. The results show clearly that water forms clusters around hydronium ion that are limited in size. These clusters also form ion pairs with the negative charge of the zeolite framework and exhibit marked fluxionality, but limited overall mobility in the zeolite channels. Water does not diffuse in the zeolite pore, but rather is primarily located in the vicinity of the BAS. This is a further structural indication that the confinement effects play a minor role in determining the acidity of the system. The limit in size is caused by the decreasing stabilization by the inner energy of the hydrogen bonded water molecules in such hydrated hydronium ions. Without these interactions water is stabilized by dispersion forces ranging between −10 and −15 kJ.mol^−1^, i.e., significantly lower than the heat of condensation of water (at ambient about 45 kJ.mol^−1^). This low enthalpic stabilization of adsorbed water without BAS or hydronium ions does not allow condensation in the constraints of the zeolite pores, as the entropy loss would be much larger than for condensation outside.

The empty space between hydrated hydronium ions is easily occupied by molecules with a higher stabilization by dispersion than that of water molecules. Sorption of neutral molecules in such an environment exerts an excess chemical potential on such molecules, which destabilizes the adsorption and eventually enhances their reactivity compared to sorption without the presence of such hydrated hydronium ions^44^. Understanding these phenomena is crucial to predict and control chemical reactivity in confines of structured hydrophilicity and hydrophobicity^45^.

Our investigation provides a detailed atomistic point of view in the open debate on the role of confinement effects in zeolites/water interfaces acidic strength. This work also defines a general computational protocol for the modeling of adsorption of organic substrates and their reactivity in zeolites exposed to high chemical potentials of water. Moreover, given the fact that these systems are considered among the most promising catalysts in the process of biomass conversion, we believe that these findings are of great importance for understanding, predicting, and controlling the catalytic activity of these peculiar interfaces.

## Methods

### Metadynamics simulations

Breaking and forming covalent bonds, such of those involved in these reactions, implies high activation energies. Transitions between different protonation states occur on a time scale that can be reached with difficulty in a standard simulation. In order to accelerate the barrier crossings, Metadynamics, an enhanced sampling method, is used to study chemical reactions. This method belongs to a class of enhanced sampling techniques based on the identification of the slow degrees of freedom involved in the reaction of interest^59,66,67^. These degrees of freedom, known in chemistry as Collective Variables, are functions of the atomic coordinates and must be chosen in order to extract the collective behavior connected to the reaction mechanism. Sampling is then accelerated by adding an external bias potential, a function of the chosen CVs, to the system potential energy. It is easy to understand how the choice of the CVs is crucial to obtain correct sampling.

In order to accelerate the exploration of every possible protonation state, as well as the diffusion of the hydronium ion inside the cavities, we adopted a recently developed set of CVs^58,68^ extremely versatile for the study of reactions involving proton transfer. A first CV, ${s}_{p}$, was used to enhance the exploration of new protonation states and push the zeolite to release a proton to the water cluster and vice versa. In this specific case, ${s}_{p}$ assumes a value close to 0 when the acidic proton is covalently bonded to the zeolite wall, +1 when complete protolysis occurs and −1 when an extra H^+^ is on the BAS site. The latter would imply a deprotonation of water to create a solvated OH^−^, which of course does not happen, see Supplementary Note 1 for details. A second CV, ${s}_{d}$, was used to explore the distance between the BAS and the hydronium accelerating the diffusion of the charge carrier inside the cavity, see Supplementary Note 1 for additional details. Born-Oppenheimer MD simulations were performed combined with Well-Tempered Metadynamics^59,60^ using PLUMED2^69^ driving the CP2K package^70^, see below.

The four zeolites, with varying cavity shape and size shown in Fig. 2, were studied loading each with an increasing number of water molecules per Brønsted acid site in a range from 1 to 8. The smallest zeolite tested, GIS-NaP1 could only accommodate up to 4 water molecules in its small cavity. The ratio Al:Si was kept constant at 1:95 which is comparable to typical experimental loadings.

### AIMD simulations

Periodic density functional theory (DFT) based AIMD were performed within the generalized gradient approximation (GGA) with the exchange correlation functional of Perdew, Burke and Ernzerhoff (PBE)^71^ and Grimme’s second-generation dispersion corrections (DFT-D2) as implemented in the CP2K package^70^. Evidences show that PBE+D2 is an excellent compromise that represents well the chemistry of not only small water clusters, but also of the extended zeolite framework and other surfaces^72–75^. Starting with the optimized zeolite unit cell with 95 SiO_2_ units and a single Al atom for each system, AIMD simulations were performed with 1, 2, 3, 4, 6 and 8 water molecule(s) at *T* = 300 K within the canonical *NVT* ensemble using a 0.5 fs time step and the CSVR^76^ thermostat to determine the local structural properties. For each simulation, well-equilibrated trajectories of ~350 *ps* were collected to obtain reliable statistical properties. For further details of the simulations and system models see Supplementary Note 2. We note that, despite the inclusion of Nuclear Quantum Effects (NQEs) may improve the accuracy of the simulations, evidence shows that larger deviations are observed at low temperature and high pressure^77^. NQEs can still change the outcomes of ambient conditions MD simulations further increasing the acidity strength of BAS. However, we expect that any contribution coming from NQEs would have shifted each trend by a similar value without changing our final conclusions.

## Supplementary information

Supplementary Information

## Data Availability

All the data and the input files necessary to reproduce the results contained in this paper are available in the Materials Cloud repository: Emanuele Grifoni, GiovanniMaria Piccini, Johannes Lercher, Vassiliki-Alexandra Glezakou, Roger Rousseau, Michele Parrinello, *Confinement effects and acid strength in Zeolites*, Materials Cloud Archive 2021.20 (2021), 10.24435/materialscloud:m8-97.
