# Supplementary material for: Confinement effects and acid strength in zeolites
Source: Nat Commun. 2021 May 11;12:2630. doi: 10.1038/s41467-021-22936-0 (PMC8113345; doi:10.1038/s41467-021-22936-0)
Supplement: Supplementary file 1 — Supplementary Information [file 41467_2021_22936_MOESM1_ESM.pdf]

# SUPPLEMENTARY INFORMATION

Emanuele Grifoni[1,2,†], GiovanniMaria Piccini[1,2,3], Johannes A. Lercher[3,4], Vassiliki-Alexandra Glezakou*[3], Roger Rousseau*[3], Michele Parrinello*[1,2,5]

[1]Department of Chemistry and Applied Biosciences, ETH Zurich, c/o USI Campus, Via Giuseppe Buffi 13, CH-6900 Lugano, Ticino, Switzerland.
[2]Institute of Computational Science, Università della Svizzera italiana (USI), Via Giuseppe Buffi 13, CH-6900, Lugano, Ticino, Switzerland.
[3]Institute for Integrated Catalysis, Pacific Northwest National Laboratory, Richland Washington, USA.
[4]Department Chemie and Catalysis Research Center, TU München, Lichtenbergstr. 4, 85747 Garching, Germany
[5]Italian Institute of Technology, Via Morego 30, 16163 Genova, Italy.

[†]Current address: Scuola Normale Superiore, Piazza dei Cavalieri, 7 I-56126 Pisa, Italy

*Corresponding authors' e-mails: vanda.glezakou@pnnl.gov, roger.rousseau@pnnl.gov, and parrinello@phys.chem.ethz.ch.

## CONTENTS

Supplementary Note 1: Collective Variables. 2
- CV1: ***sp***. 2
- CV2: ***sd***. 4
- Restraint: ***sr***. 5

Supplementary Note 2: Ab-initio MD setup. 7
- Cell parameters. 7
- Samples preparation. 7
- Simulation lengths. 8

Supplementary Note 3: Well-Tempered Metadynamics setup. 8
Supplementary Note 4: Water cluster analysis. 8
- 4.1: Al-$O_w$ radial distribution functions. 8
- 4.3: Water cluster sphericities. 11

Supplementary Note 5: Validation of interatomic potential. 12

## Supplementary Note 1: Collective Variables.

In Voronoi diagrams, volume is partitioned in polyhedra around points called seeds or generators. Every polyhedron is defined as the portion of volume whose points are closer to a specific seed than to any other. Similarly, hydrogen atoms assigned to the site $i$ are taken as the fraction of protons that are much closer to this site than from all the other ones. Softmax functions allow to reproduce this behaviour without any discontinuity or singularity as shown in the **Eqs. (**1**)** and **(**2**)**,

$$\omega_i(\mathbf{r}) = \frac{e^{-\lambda|\mathbf{R}_i - \mathbf{r}|}}{\sum_m e^{-\lambda|\mathbf{R}_m - \mathbf{r}|}}, \quad (1)$$

$$\rho_i = \sum_{j \in H} \omega_i\,(\mathbf{R}_j), \quad (2)$$

where $\mathbf{R}$ is a vector of the atomic positions, the indexes $i$ and $m$ run over the atoms able to bond or release hydrogen atoms, $j$ runs over the hydrogen atoms and $\lambda$ is a parameter that controls steepness and selectivity of this function. $\omega_i(r)$ approaches 1 when the atom $i$ is the closest to $r$ and 0 otherwise, see **Supplementary Figure 1**.

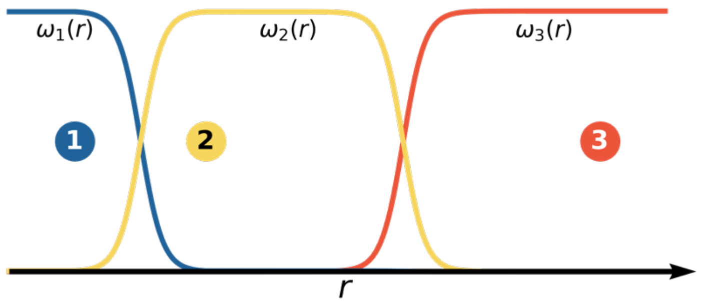


**Supplementary Figure 1** 1D example of the **Eq. (**1**)**

Finally, the summation over the hydrogen atoms returns $\rho_i$, the total number of protons within the $i$th voronoi polyhedron.

### CV1: $s_p$.

Once we have obtained the instantaneous number of hydrogen atoms around each site, the difference between these values, $\rho_i$, and the respective references, $\rho_i^0$, returns their deviation from neutrality, see

**Eq. (3)**. Then, the overall excess or defect of protons for every moiety is taken as the summation of their site contributions, see **Eq. (4)**.

$$\delta_i = \rho_i - \rho_i^0 \qquad (3)$$

$$q_k = \sum_{i \in k} \delta_i \qquad (4)$$

In this specific case, $q_0$ describes the overall excess or defect of protons of the BAS and is obtained by summing all the $\delta_i$ with $i$ that runs over each BAS oxygen atom. Similarly, $q_1$ describes the overall excess or defect of protons of the water clusters and it is obtained by summing all the $\delta_i$ with $i$ that runs over each water oxygen atoms.

The result of this operation is a vector $\vec{q} = (q_0, q_1)$ that describes with its components the protonation state of our system. In order to further reduce the dimensionality of the problem, we project this vector into a single a scalar value. This is done by linearly combining $q_0$ and $q_1$ as in **Eq. (5)**.

$$s_p = 1 \cdot q_0 + 2 \cdot q_1 \qquad (5)$$

In systems such as ours characterized by two different inequivalent moieties able to exchange protons (BAS and water cluster), in principle three theoretical protonation states are possible, see **Supplementary Table 1**. Assuming we do not know anything about its reactivity, every chemical group can donate and accept a hydrogen atom.

**Supplementary Table 1** The two components of the vector $\vec{q}$ and the respective $s_p$ values.

| $q_0$ | $q_1$ | | $1 \cdot q_0$ | $2 \cdot q_1$ | | $s_p$ |
|---|---|---|---|---|---|---|
| 0 | 0 | | 0 | 0 | | 0 |
| +1 | -1 | $\Rightarrow$ | +1 | -2 | $\Rightarrow$ | -1 |
| -1 | -1 | | -1 | +2 | | +1 |

As we said, $q_0$ and $q_1$ have been chosen in order to represent the overall deviation from neutrality of BAS and water clusters respectively. Since the BAS cannot act like a base and therefore cannot gain a proton from the water cluster, the state described by $\vec{q} = (+1, -1)$ is totally unphysical and $s_p$ will

never reach negative values. Then, $s_p = 0$ $(\overrightarrow{q} = (0,0))$ describes the undissociated states, while $s_p = 1$ $(\vec{q} = (-1, +1))$ describes the states in which a proton is transferred from the BAS to the water cluster.

This CV ensures the possibility to explore every protonation states starting from the most energetically accessible up to the highest one in energy without imposing any restriction.

## CV2: $s_d$.

With this CV we measure the distance between the sites that have exchanged a proton and, thus, not lying in their reference states.

$$s_d = -\sum_{i,m>i} r_{im} \cdot \delta_i \cdot \delta_m \qquad (6)$$

Here the reference value of protons in each Voronoi polyhedron is taken as the total number of protons assigned to the entire group $N_{H\in k}$ divided by the the total number of sites belonging to the $k$-th group, $N_k$. Starting from the **Eq. (2)**, the instantaneous deviation from its reference is computed as follow:

$$\delta_i = \rho_{i\in k} - \frac{N_{H\in k}}{N_k}. \qquad (7)$$

In the example of a BAS and 4 water molecules, let us take the protolysis of the BAS and consequent protonation of a water molecule. The water oxygen atoms have $N = 4$ and $N_H = 8$ while the four BAS oxygen atoms have $N = 4$ and $N_H = 1$. Before the reaction has taken place, every water oxygen atom has a value of $\rho$ approximately equal to 2, $\frac{N_H}{N} = 2$ and therefore $\delta \approx 0$. After having subtracted a proton by the BAS, one of the water oxygen sites will have $\rho = 3$ and then $\delta = 1$. The same operation can be done for the four oxygen atoms of the BAS. Before the protolysis, assuming the hydrogen atom is bonded to the first oxygen, the four sites have $\rho_0 = 1$, $\rho_1 = 0$, $\rho_2 = 0$ and $\rho_3 = 0$. Then their values of $\delta$ are +0.75, -0.25, -0.25 and -0.25 respectively. After the reaction has taken place all sites have $\delta$ equal to -0.25. This ensures that the opposite sign terms give a zero contribution in the undissociated case (**Supplementary Figure 1a**) and an averaged one in the other (**Supplementary Figure 1b**).

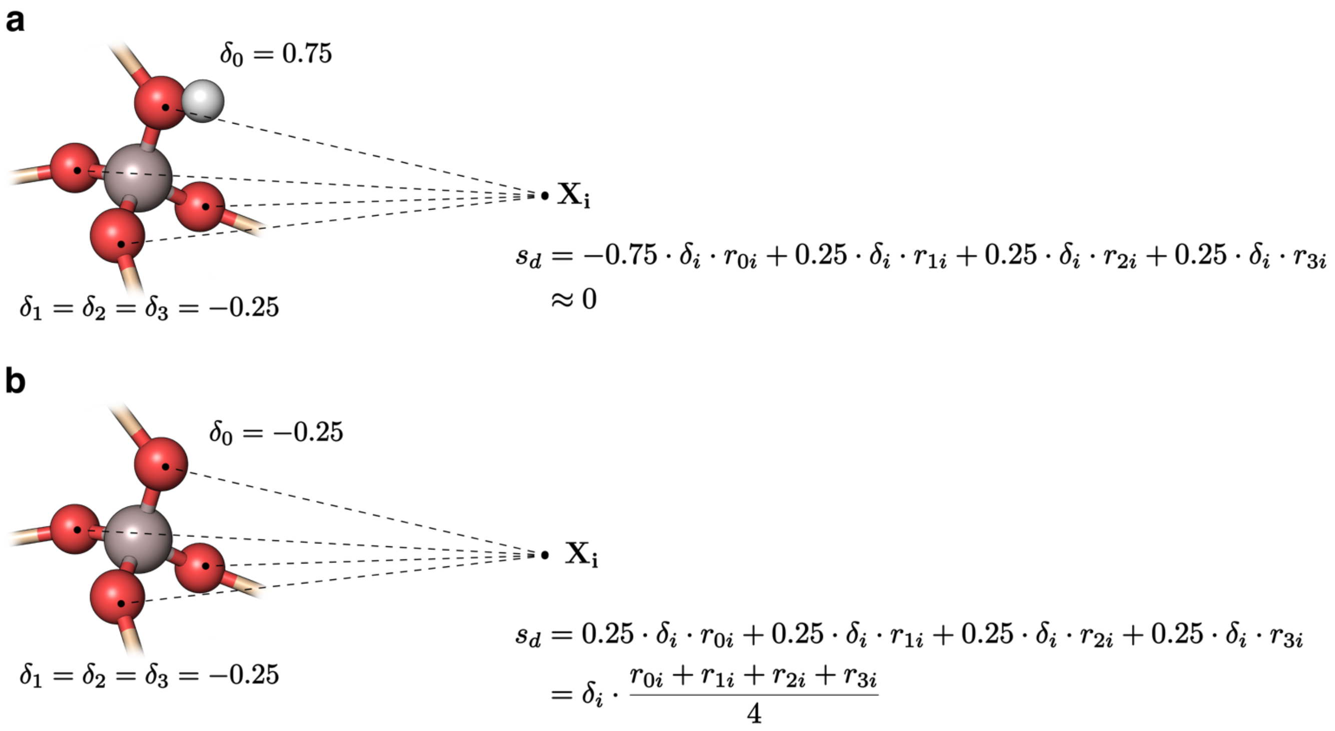


**Supplementary Figure 1** Schematic view of $s_d$ calculation between protonated (a) or deprotonated (b) BAS, and a generic species $X_i$.

## Restraint: $s_r$.

In order to prevent simultaneous dissociation events, we restraint a third CV used only to monitor how many sites out of their reference states are present. The functional form of this CV is:

$$s_r = \sum_i \sqrt{\delta_i^2 + \alpha^2}, \qquad (8)$$

where $i$ runs over the site indexes and $\alpha$ is a positive number much less than 1. With a proper value of $\alpha$ the square root term is a good approximation of the absolute value that allows to avoid the singularity for $\delta_i = 0$ (see **Supplementary Figure 2**). This CV returns the summation of the partial charge moduli and, by restraining it, we can limit at the given time the number of reacted pairs simultaneously present.

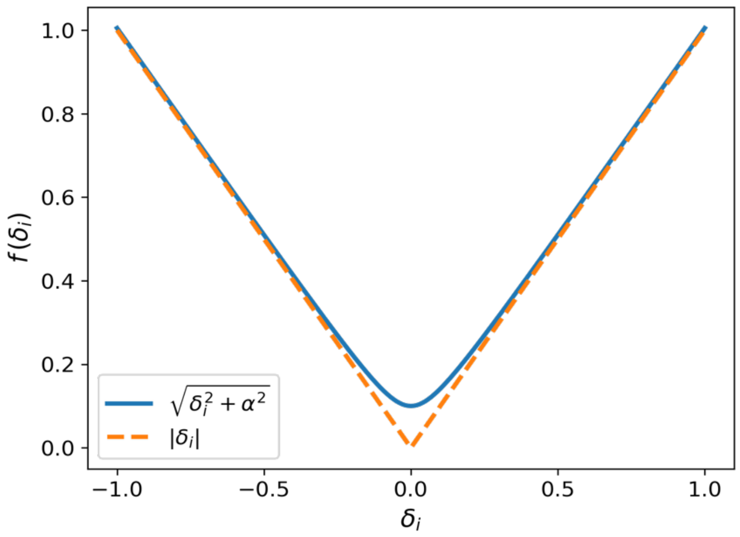


**Supplementary Figure 2** Different behavior of absolute value function (orange dashed line) and the smoothed version (blue line) in proximity of $\delta_i$. The parameter α controls the smoothness of the curve. In this plot the value of α has been set equal to 0.1.

## Supplementary Note 2: Ab-initio MD setup.

All the simulations have been performed with CP2K package[1] and set up as reported in **Supplementary Table 2**.

**Supplementary Table 2** Ab initio MD parameters.

| | |
|---|---|
| Ensemble | NVT |
| Temperature (K) | 300 |
| Thermostat | Canonical Sampling through Velocity Rescaling[2] |
| Basis sets | DZVP-MOLOPT-SR-GTH[3] |
| Potential | GTH-PBE[4] |
| Energy cutoff (Ry) | 280 |
| Relative cutoff (Ry) | 40 |
| EPS SCF | 1.0E-6 |
| XC Functional | PBE[5] |
| Time step (fs) | 0.5 |

## Cell parameters.

| | | | | |
|---|---|---|---|---|
| **CHA** | $A, B, C$ | 18.918 | 18.918 | 18.918 |
| | $\alpha, \beta, \gamma$ | 94.07 | 94.07 | 94.07 |
| **MFI** | $A, B, C$ | 20.4722 | 20.1087 | 13.5758 |
| | $\alpha, \beta, \gamma$ | 89.9710 | 89.8752 | 89.9915 |
| **FAU** | $A, B, C$ | 17.698 | 35.396 | 17.698 |
| | $\alpha, \beta, \gamma$ | 60.0 | 60.0 | 60.0 |
| **GIS** | $A, B, C$ | 29.403 | 19.602 | 10.1580 |
| | $\alpha, \beta, \gamma$ | 90.0 | 90.0 | 90.0 |

## Samples preparation.

Each system has a composition of 96 units of $SiO_2$ with an aluminum atom replacing a silicon. All of them have been thermalized with a 5 ps NVT MD simulation.

## Simulation lengths.

**Supplementary Table 3** Total simulation lengths in ps.

| | $1 \cdot H_2O$ | $2 \cdot H_2O$ | $3 \cdot H_2O$ | $4 \cdot H_2O$ | $6 \cdot H_2O$ | $8 \cdot H_2O$ |
|---|---|---|---|---|---|---|
| **CHA** | 706.594 | 381.480 | 317.026 | 172.475 | 240.444 | 246.292 |
| **MFI** | 702.039 | 304.055 | 577.134 | 693.118 | 361.787 | 226.910 |
| **FAU** | 234.212 | 173.790 | 233.670 | 215.216 | 175.632 | 121.998 |
| **GIS** | 244.048 | 240.732 | 212.111 | 194.855 | - | - |

# Supplementary Note 3: Well-Tempered Metadynamics setup.

Parameters adopted for PLUMED2[6,7] settings are reported in **Supplementary Table 4**.

**Supplementary Table 4** PLUMED parameters.

| | |
|---|---|
| Gaussian hills heights | 0.35 |
| Gaussian hills widths ($s_p$) | 0.2 |
| Gaussian hills widths ($s_d$) | 0.4 |
| Bias factor | 10 |
| Temperature (K) | 300 |
| Hills deposition rate | 100 |
| $\lambda$ ($s_p$) | 5 |
| $\lambda$ ($s_d$) | 8 |
| $\lambda$ ($s_r$) | 8 |
| $\alpha$ ($s_r$) | 1.0E-4 |

# Supplementary Note 4: Water cluster analysis.

## 4.1: Al-$O_w$ radial distribution functions.

**Supplementary Figure 3** and **Supplementary Figure 4** show how water molecules are distributed around the BAS site of each zeolite and at every level of water loading. Radial distribution functions and their integrals over distance show that water clusters are arranged around the BAS using roughly the same portion of volume irrespective the framework type.

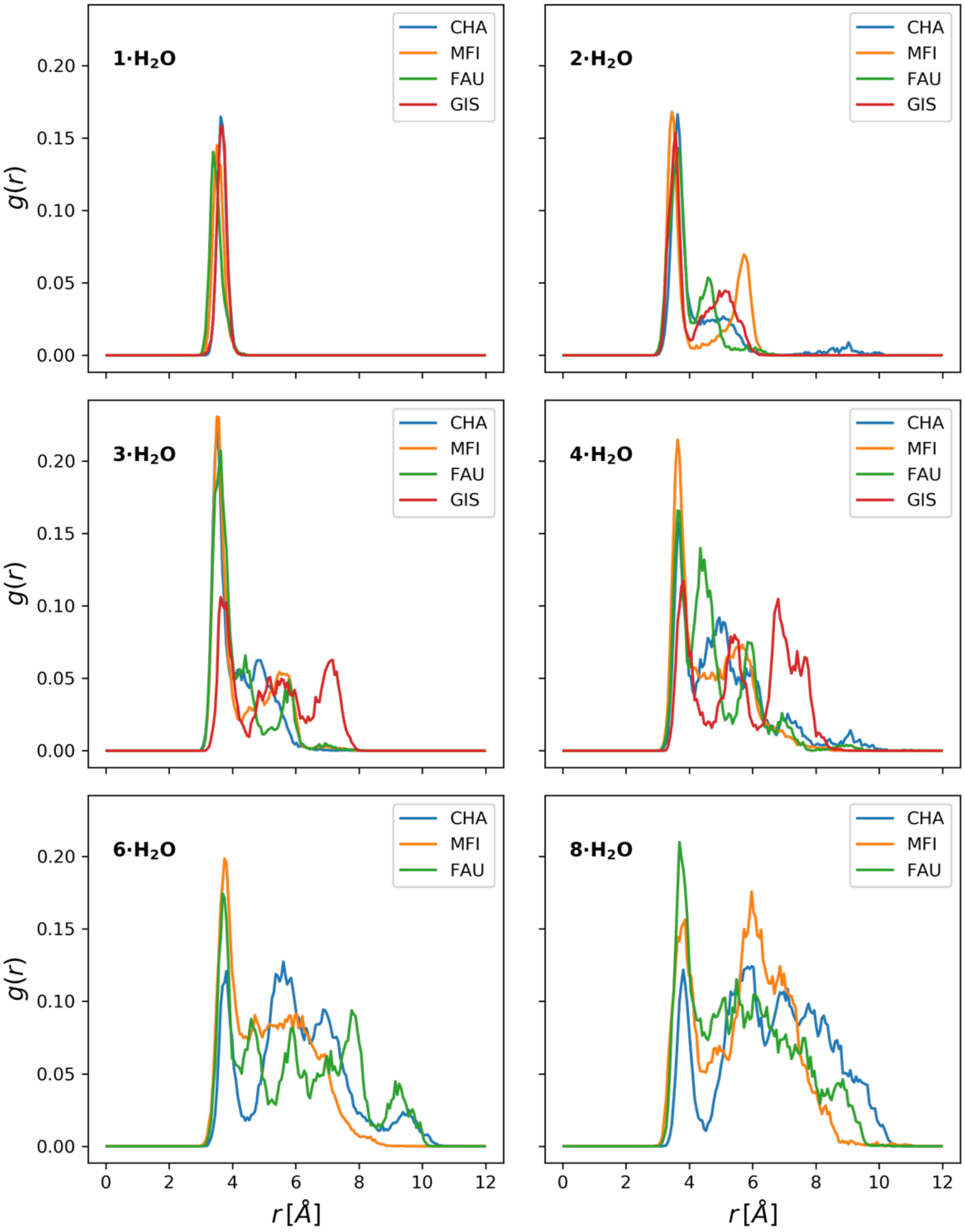


**Supplementary Figure 3** Al-$O_w$ radial distribution functions.

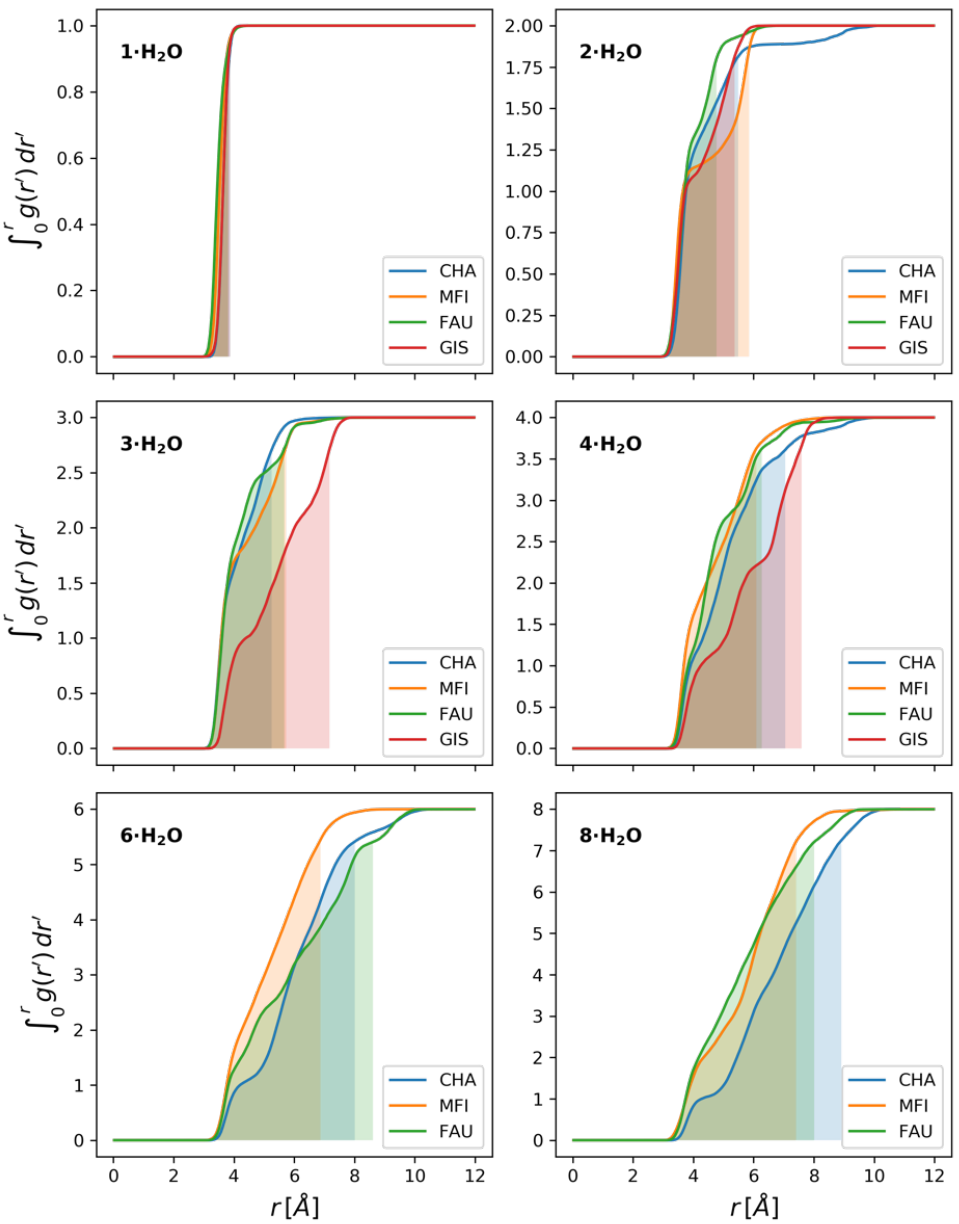


**Supplementary Figure 4** Integrals of radial distribution functions of water along $r$. The filled areas indicate the radius of the sphere centered on the aluminum atom and whose surfaces enclose 90% of their distribution functions, $g(r)$.

## 4.3: Water cluster sphericities.

An indication that the framework does not affect the water cluster behavior comes from the analysis of their shapes. A volume and a surface can be computed for polyhedra whose vertices are defined by 4 or more water molecules. From these we can get an estimation of their sphericity according to the Wadell definition[8]:

$$\psi_p = \frac{\pi^{\frac{1}{3}}(6V_p)^{\frac{2}{3}}}{A_p}. \quad (1)$$

This formula defines the sphericity, $\psi_p$, of a generic polyhedron as the ratio between its volume $V_p$ and its surface $A_p$. $\psi_p$ can assume values between 0 and 1, where, by definition, the sphericity of the cluster is unity for a sphere and decreases in solids with lower symmetry. The sphericities of these clusters inside the zeolites were calculated and compared with the values extracted from the equivalent MD simulations in gas phase, see **Supplementary Table 5**. Results show that water clusters confined in zeolite frameworks are not significantly impacted by a changing confinement and they have values comparable with the gas-phase ones.

**Supplementary Table 5** Water clusters sphericity, $\psi$, into four different zeolite cavities and in gas phase (GP).

| $n_{H_2O}$ | $\psi_{GP}$ | $\psi_{CHA}$ | $\psi_{MFI}$ | $\psi_{FAU}$ | $\psi_{GIS}$ |
|---|---|---|---|---|---|
| 4 | 0.34 ± 0.15 | 0.33 ± 0.15 | 0.39 ± 0.14 | 0.45 ± 0.11 | 0.37 ± 0.14 |
| 6 | 0.68 ± 0.08 | 0.64 ± 0.08 | 0.69 ± 0.10 | 0.52 ± 0.14 | - |
| 8 | 0.78 ± 0.07 | 0.74 ± 0.08 | 0.74 ± 0.05 | 0.66 ± 0.07 | - |

## Supplementary Note 5: Validation of interatomic potential.

Errore. L'origine riferimento non è stata trovata. shows the Ow-Ow radial distribution functions of gas p hase water clusters simulated with PBE+D2 and B3LYP+D2 exchange and correlation functionals. This analysis proved that our structures are in agreement with the ones described by a higher level of theory, in that they give identical peak maxima for both first and second shells, and that the over-binding errors due to the PBE are well compensated by the Grimme dispersion corrections.

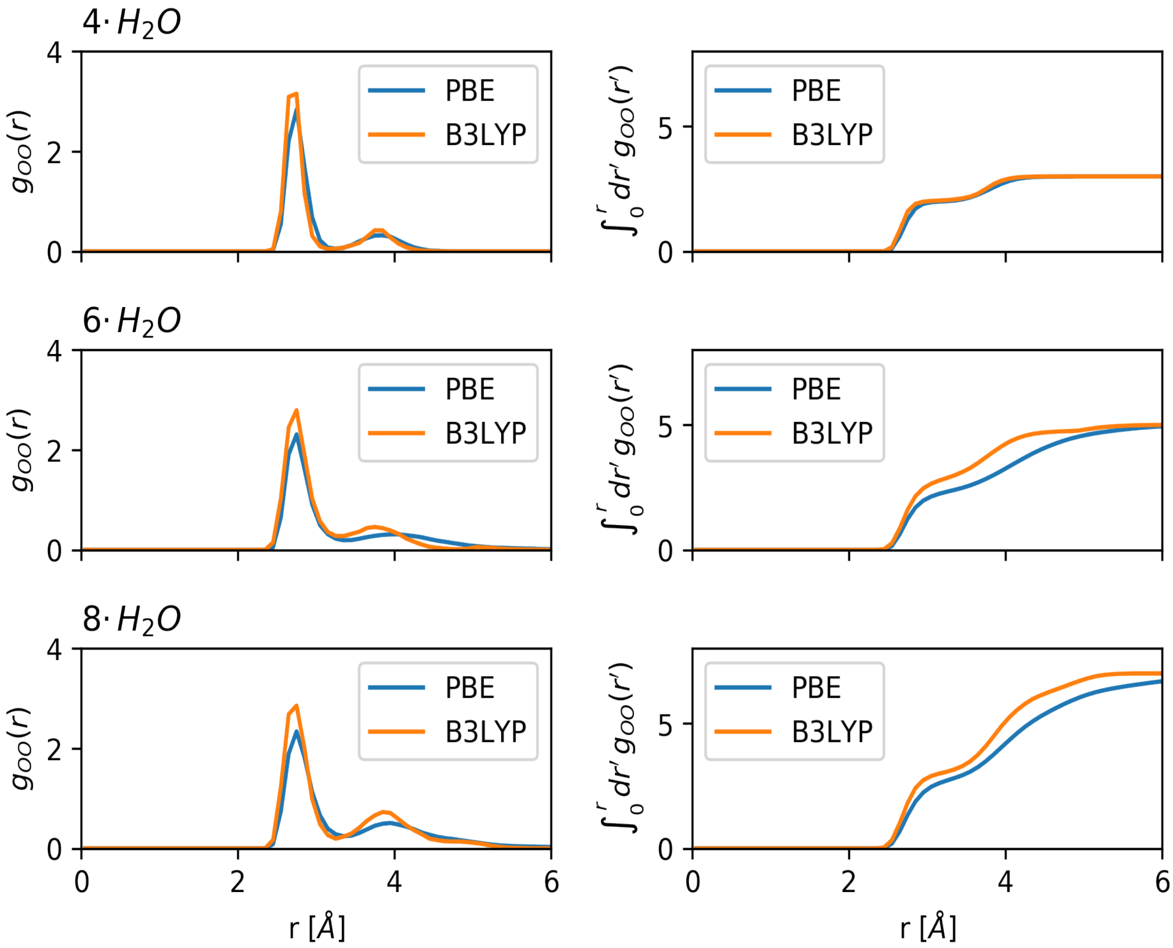


**Supplementary Figure 5** O-O radial distribution functions and their integrals over distances. Blue lines report water clusters behavior simulated with PBE functional, while orange lines report the same results performed with B3LYP functional.

# Supplementary References


(1) Vandevondele, J.; Krack, M.; Mohamed, F.; Parrinello, M.; Chassaing, T.; Hutter, J. Quickstep: Fast and Accurate Density Functional Calculations Using a Mixed Gaussian and Plane Waves Approach. *Comput. Phys. Commun.* **2005**, *167* (2), 103–128. https://doi.org/10.1016/j.cpc.2004.12.014.

(2) Bussi, G.; Donadio, D.; Parrinello, M. Canonical Sampling through Velocity Rescaling. *J. Chem. Phys.* **2007**, *126* (1). https://doi.org/10.1063/1.2408420.

(3) VandeVondele, J.; Hutter, J. Gaussian Basis Sets for Accurate Calculations on Molecular Systems in Gas and Condensed Phases. *J. Chem. Phys.* **2007**, *127* (11). https://doi.org/10.1063/1.2770708.

(4) Goedecker, S.; Teter, M.; Hutter, J. Separable Dual-Space Gaussian Pseudopotentials. *Phys. Rev. B* **1996**, *54* (3). https://doi.org/10.1103/PhysRevB.54.1703.

(5) Perdew, J. P.; Burke, K.; Ernzerhof, M. Generalized Gradient Approximation Made Simple. *Phys. Rev. Lett.* **1996**, *77* (18), 3865–3868. https://doi.org/10.1103/PhysRevLett.77.3865.

(6) Tribello, G. A.; Bonomi, M.; Branduardi, D.; Camilloni, C.; Bussi, G. PLUMED 2: New Feathers for an Old Bird. *Comput. Phys. Commun.* **2014**, *185* (2), 604–613. https://doi.org/10.1016/j.cpc.2013.09.018.

(7) Bonomi, M.; Bussi, G.; Camilloni, C.; Tribello, G. A.; Banáš, P.; Barducci, A.; Bernetti, M.; Bolhuis, P. G.; Bottaro, S.; Branduardi, D.; et al. Promoting Transparency and Reproducibility in Enhanced Molecular Simulations. *Nat. Methods* **2019**, *16* (8), 670–673. https://doi.org/10.1038/s41592-019-0506-8.

(8) Wadell, H. Volume, Shape, and Roundness of Quartz Particles. *J. Geol.* **1935**, *43* (3), 250–280. https://doi.org/10.1086/624298.

(9) Becke, A. D. Density-functional Thermochemistry. III. The Role of Exact Exchange. *J. Chem. Phys.* **1993**, *98* (7), 5648–5652. https://doi.org/10.1063/1.464913.

(10) Brandenburg, J. G.; Hochheim, M.; Bredow, T.; Grimme, S. Low-Cost Quantum Chemical Methods for Noncovalent Interactions. *J. Phys. Chem. Lett.* **2014**, *5* (24), 4275–4284. https://doi.org/10.1021/jz5021313.
